# Edge Exposure and Electrochemical Modulation of Graphene Oxide by Focused Cu Ion Bombardment

**DOI:** 10.1021/acs.langmuir.6c01117

**Published:** 2026-06-23

**Authors:** Jan Luxa, Petr Malinský, Josef Novák, Vladimír Havránek, Vlastimil Mazánek, Jakub Regner, Zdeněk Sofer

**Affiliations:** † Department of Inorganic Chemistry, 52735 University of Chemistry and Technology Prague, Technická 5, Prague 6 166 28, Czech Republic; ‡ Nuclear Physics Institute of the Czech Academy of Sciences, Hlavní 130, Řež 250 68, Czech Republic; § Department of Physics, Faculty of Science, University of J. E.Purkyně, Usti Nad Labem 400 96, Czech Republic

## Abstract

The modification of graphene oxide by energetic ion beams offers a promising alternative to conventional reduction techniques, providing spatially controlled tuning of the structural and electrochemical properties. In this study, we investigate the effect of focused Cu ion beam irradiation on free-standing graphene oxide foils using a comprehensive suite of characterization methods, including scanning electron microscopy, energy-dispersive spectroscopy, Raman spectroscopy, atomic force microscopy, X-ray photoelectron spectroscopy, and scanning electrochemical microscopy. Cu ion bombardment was found to induce partial reduction of graphene oxide, evidenced by a decrease in oxygen content, an increased C/O ratio, and enhancement of graphitic carbon features. Scanning electrochemical microscopy measurements using the [Ru­(NH_3_)_6_]^3+^ redox probe revealed significantly enhanced electrochemical activity in and around the irradiated regions. This enhancement is likely influenced by local thinning of the graphene oxide layer and the increased exposure of reactive edge regions generated during irradiation rather than adsorption effects being the dominant factor. Elemental mapping confirmed a depletion of the Ru signal within bombarded stripes, supporting the structural origin of activity enhancement. These findings establish Cu-ion irradiation as a reagent-free, maskless approach for nanoscale patterning of electrochemically active regions in graphene oxide, with potential applications in sensing, catalysis, and electronic devices.

## Graphical abstract

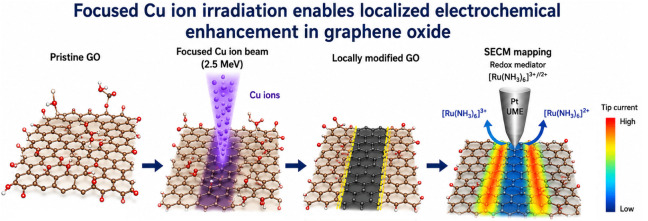

## Introduction

2D layered materials have been at the forefront of material research ever since the discovery of graphene in 2004.^1^ Graphene oxide (GO) is one of the most studied functional derivatives of graphene with a wide range of applications in various fields. With various functional groups such as hydroxy, epoxy, or carboxy groups GO’s properties can be fine-tuned for applications in electronics, electrochemistry, biomedicine, and others.^2–6^ Another key factor for GO is the possibility to tune its properties using various techniques such as chemical or thermal reduction, functionalization, or, for example, ion bombardment.^6–8^

Modifying GO properties via controlling the oxygen content seems particularly interesting, as, for example, electrical conductivity or electrochemical activity are proportional to the conductivity of GO.^9,10^ However, the traditional methods (chemical and thermal reduction) lack the precision necessary for precisely distinguishing the differences in the properties of pristine GO and reduced GO (rGO).^11^ Using alternative methods, such as energetic ion beams, might enable targeted modifications of surface composition and structure.^12^ By selectively removing the oxygen functional groups via mask bombardment or direct beam writing, ion bombardment may serve to provide a controllable approach to fine-tune GO’s properties, particularly in electrochemical applications.^12,13^ However, the impact of such modifications by energetic ion beams on electrochemical properties and patterning remains to be studied.

The interaction of GO with energetic ion beams revolves around a complex interplay of electronic and nuclear energy-loss mechanisms.^14^ When high-energy ions penetrate the GO matrix, they transfer energy through electronic excitation and atomic collisions. This energy transfer leads to the removal of oxygen functional groups, reducing the level of GO to a more graphene-like structure. The extent of reduction depends on many factors such as ion species, their energy, and fluence.^14–16^ In general, more energetic ions lead to greater oxygen removal and improved restoration of the sp^2^ carbon network. The effects of both electronic energy loss as well as nuclear energy loss play an important role in modifying the material, where higher electronic energy losses contribute to defect healing and structural restoration, while nuclear energy losses facilitate the removal of oxygen functionalities.^7,17–20^ Studies have shown that ion bombardment can selectively modify the surface structure while maintaining the bulk integrity of the material, making it a promising technique for controlled GO reduction.^20–23^

The influence of various ions on GO’s properties has been reported in the literature. Experiments with 500 keV Ar^+^, 1 MeV Si^+^, and 5 MeV Au^+^ ions have demonstrated that ion energy and fluence significantly influence the degree of GO reduction.^21,22,24^ Furthermore, helium and hydrogen ion irradiation at MeV energies has been found to effectively reduce GO, with He showing greater effect due to its higher electronic energy loss.^17^ Similarly, Au ions with high energy and fluence have resulted in extensive oxygen removal, leading to a highly reduced and electrically conductive material.^7,20,25^ Importantly, studies also indicate that ion beam irradiation can achieve reduction comparable to or better than traditional chemical or thermal methods, while avoiding toxic reagents or extreme processing conditions.^26,27^ These findings highlight the potential of ion beam irradiation as an alternative and environmentally friendly method for tailoring GO’s properties.

Here, we report a systematic investigation of the effect of Cu-ion-beam irradiation on free-standing graphene oxide foils and correlate the induced structural changes with variations in local electrochemical activity. Copper ions were selected due to their intermediate mass and high electronic stopping power, offering a unique balance between nuclear and electronic interactions when compared with lighter ions like helium or hydrogen and heavier species such as gold. Additionally, copper holds practical relevance for micro- and nanoscale patterning in flexible and functional electronic devices.

To study the impact of Cu irradiation, we employed a comprehensive suite of characterization techniques, including scanning electron microscopy, energy-dispersive X-ray spectroscopy, Raman spectroscopy, atomic force microscopy, X-ray photoelectron spectroscopy, and scanning electrochemical microscopy. The electrochemical measurements were performed using the outer-sphere redox mediator [Ru­(NH_3_)_6_]­Cl_3_, which enables probing of local electron-transfer behavior and irradiation-induced electronic modifications.

This integrated approach enabled us to observe morphological and compositional modifications in the irradiated regions, which appeared as stripe-like features with a reduced oxygen content and localized thinning of the GO layer. Chemical analysis revealed a higher carbon-to-oxygen ratio and spectroscopic signatures consistent with the partial reduction of the GO. Electrochemical mapping showed enhanced activity within and around the bombarded regions, which we attribute to the increased exposure of reactive GO edge sites created by structural damage or material removal. Importantly, elemental mapping demonstrated that ruthenium species did not preferentially accumulate on the irradiated areas, suggesting that adsorption is unlikely to be the dominant origin of the enhanced electrochemical response and that irradiation-induced structural and electronic modifications of GO likely play a significant role.

These findings position Cu-ion-beam irradiation as a maskless, reagent-free strategy for spatially tuning the properties of GO, with potential applications in the fabrication of advanced electrochemical devices, microsensors, and patterned catalytic platforms.

## Results and Discussion

A graphene oxide foil was modified using a focused ion beam utilizing 2.5 MeV Cu^4+^ ions with an ion fluence of 1 × 10^14^ cm^–2^. Schematically, the experimental setup is shown in Figure 1a. Figure 1b shows that the ion beam was used to create stripes with various widths ranging from 10 to 40 μm, a gap of 60 μm, and a stripe length of 500 μm.

**Figure 1:**
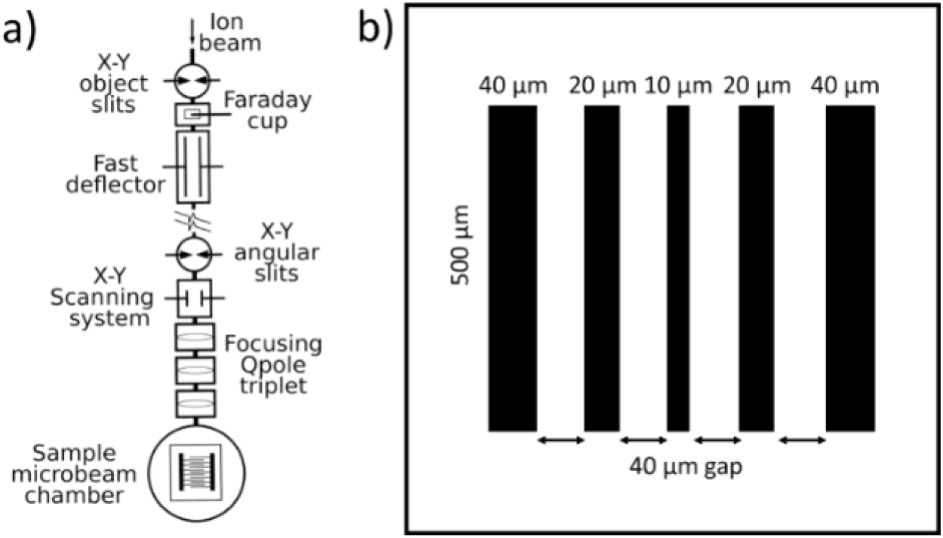
a) A schematic illustration of the maskless production of microstructures by ion beam writing. b) Illustration of the microstructures prepared by copper ion beam writing on the surface of GO.

The morphology of the irradiated GO foil was first studied by scanning electron microscopy, as displayed in Figure 2a. The micrograph highlights the bombarded areas as dark stripes with various widths and a spacing of 60 μm. Additionally, we observed cracks in a close proximity to the stripes, which likely originate from the heat transfer during ion bombardment, resulting in GO cracking.^28^ Energy-dispersive spectroscopy was also performed, with results shown in Figure 2b to Figure 2d.

**Figure 2:**
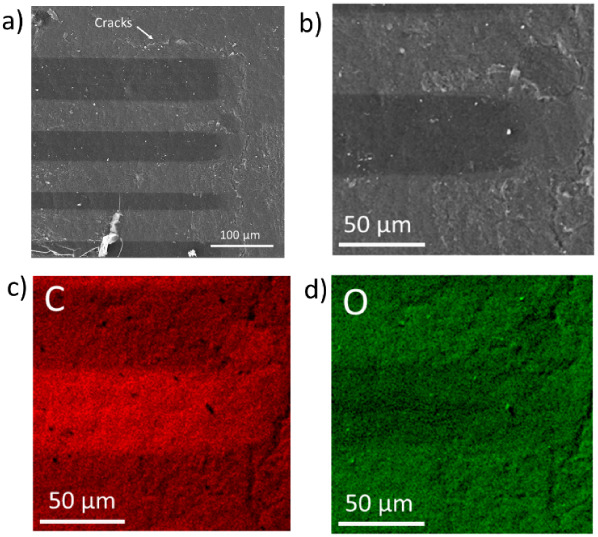
a) SEM micrograph of Cu-bombarded GO foil showing the bombarded stripes and cracks. b) Image from where EDS was taken. c) EDS elemental map of carbon and d) oxygen.

The elemental map of carbon shows an increased carbon content within the stripe, which is due to partial carbonization of the GO foil induced by the ion bombardment.^29^ In contrast, the oxygen elemental maps highlight the decrease in oxygen content in the irradiated area, indicating a reduction of oxygen functionalities. These results are in good agreement with previous reports on ion-irradiated GO materials.^30^

The Raman spectra of pristine and Cu-bombarded GO foils are shown in Figure 3a. Both spectra exhibit the characteristic D (∼1350 cm^–1^) and G (∼1580 cm^–1^) bands commonly observed in graphene oxide materials.^31–33^ The D band originates from defect-activated scattering associated with disorder in the graphitic lattice, while the G band corresponds to the in-plane vibrational mode of sp^2^-bonded carbon atoms. The intensity ratio between these bands (I_D_/I_G_) is commonly used as an indicator of structural disorder and defect density in carbon-based materials.^34^ The pristine GO foil exhibited an I_D_/I_G_ ratio of 1.01 ± 0.07, whereas the Cu-bombarded region showed a reduced ratio of 0.88 ± 0.05. In the region containing cracks adjacent to the irradiated stripes, the I_D_/I_G_ ratio remained comparable to pristine GO (0.97 ± 0.09), suggesting that the structural modifications in these regions were less pronounced. Such changes in the irradiated areas indicate modification of graphitic ordering and defect structure following ion irradiation, which is also consistent with the XPS results discussed in the following sections.

**Figure 3:**
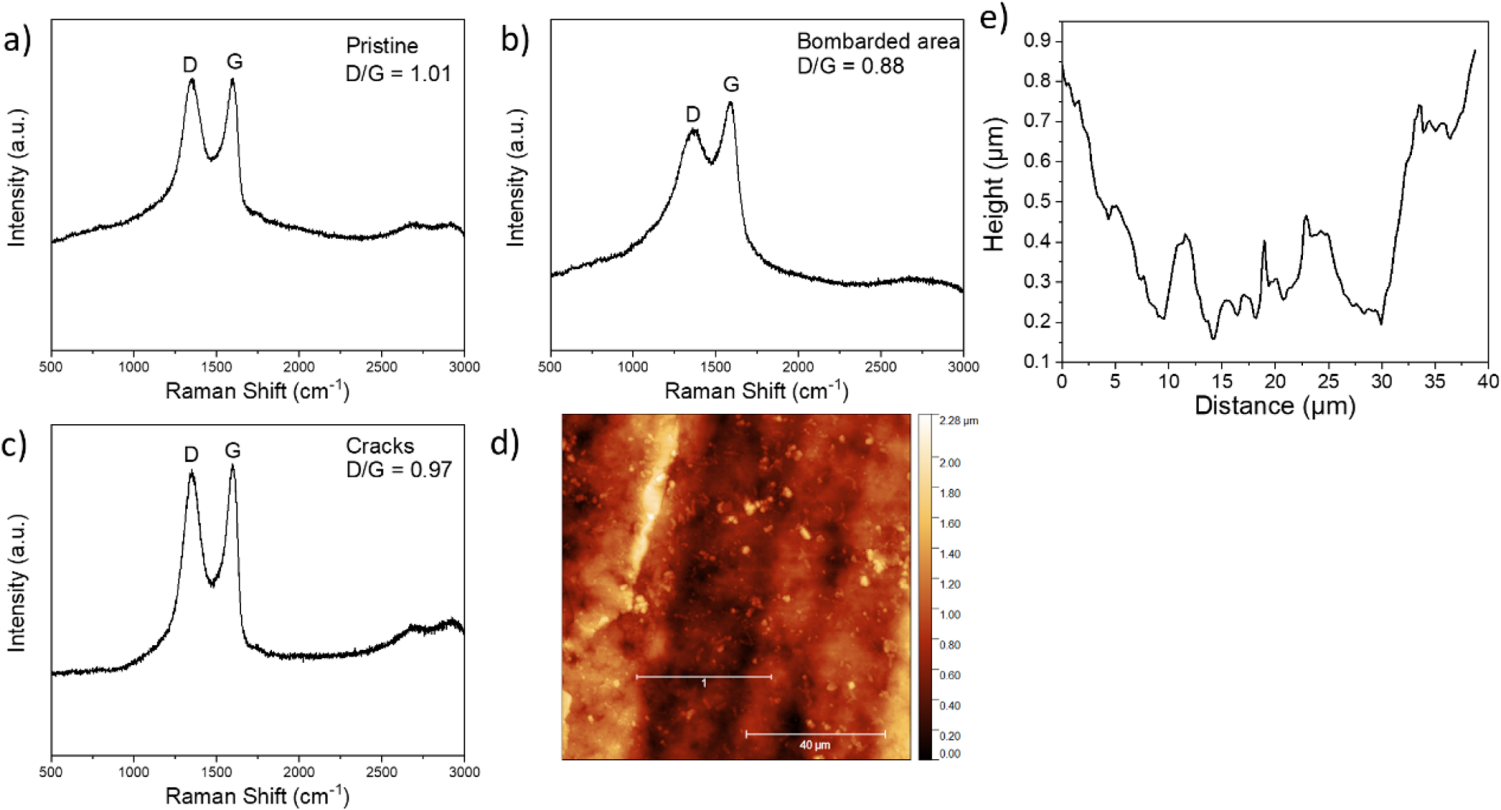
a) Raman spectrum of pristine GO foil, b) Raman spectrum of GO foil bombarded by Cu ions, c) Raman spectrum of the cracks around the irradiated area, d) AFM of GO foil with the irradiated area, and e) height profile extracted from the AFM measurements.

The GO foil was also characterized by atomic force microscopy (AFM), with results shown in Figure 3d and a height profile across the bombarded stripe shown in Figure 3e. The height profile shows that the stripe with a width of 20 μm was analyzed and that in comparison with the pristine GO foil, its depth was around 0.6–0.7 μm. The decrease in the height of the GO foil after ion bombardment could be caused by the removal of atoms from the surface and/or the removal of oxygen functional groups, which is consistent with Raman spectroscopy and EDS results indicating changes in graphitic ordering together with reduced oxygen content in the bombarded regions

To further investigate the influence of Cu ion bombardment on the chemical composition of GO, X-ray photoelectron (XPS) measurements were conducted, as shown in Figure 4a–e. Since the ion-bombarded stripes were too small for localized XPS analysis, an additional GO foil bombarded under identical irradiation conditions over a larger area (∼1 cm^2^) was prepared to enable a more reliable assessment of the surface chemical composition. Although local variations associated with beam overlap and edge effects may exist in the microstructured stripes used for SECM, the larger area-irradiated sample is expected to reflect the dominant chemical trends induced by Cu ion bombardment under identical irradiation conditions.

**Figure 4:**
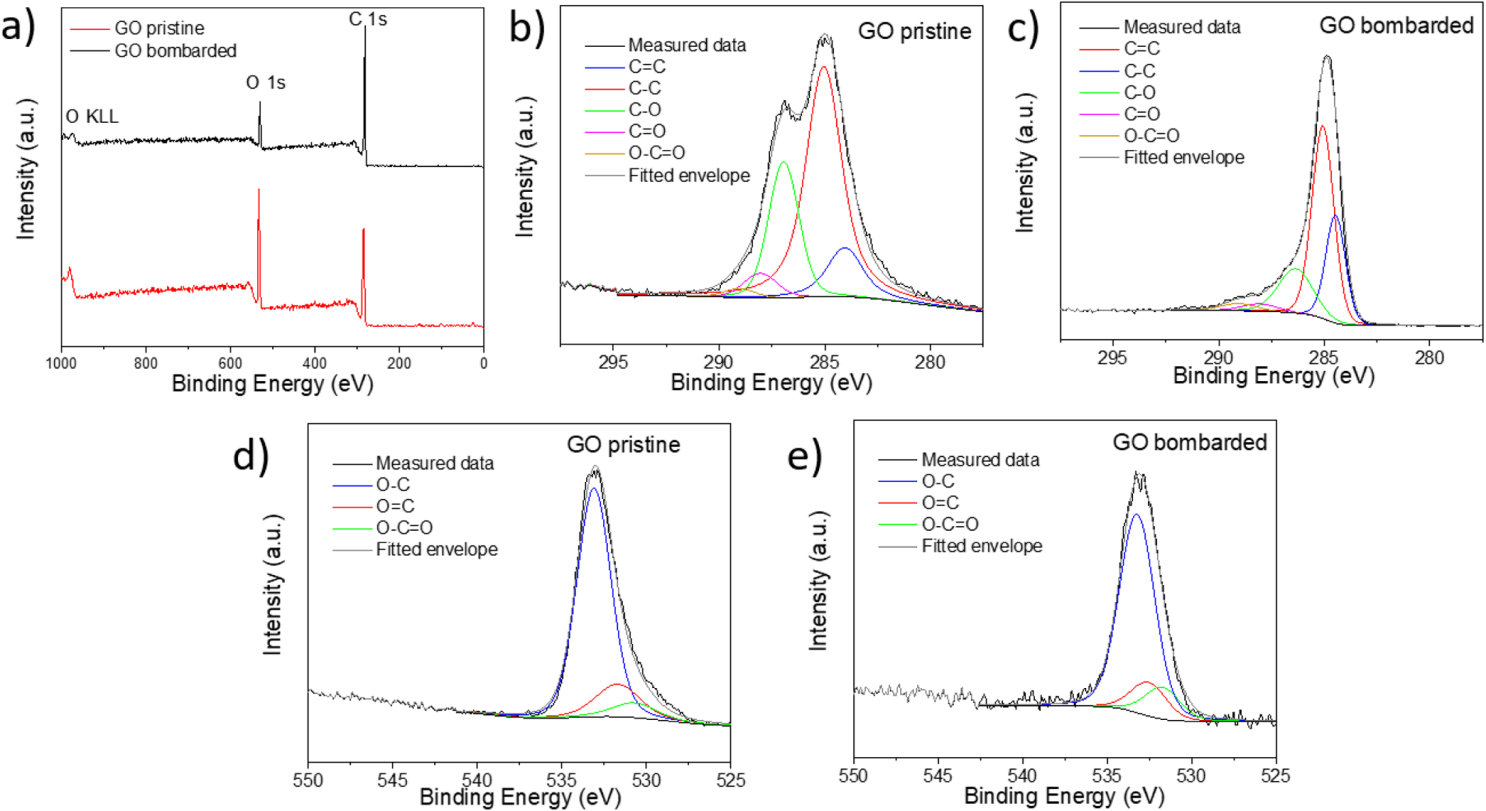
a) Survey spectra of pristine and bombarded GO foils, b) C 1s region of pristine GO foil, c) C 1s region of bombarded GO foil, d) O 1s region of pristine GO foil, and e) O 1s region of bombarded GO foil.

The survey spectra shown in Figure 4a reveal a substantial increase in the C/O atomic ratio from 2.96 for pristine GO to 7.68 after Cu-ion bombardment. Although the C/O ratio alone cannot be considered a definitive indicator of GO reduction due to the possible presence of adsorbed oxygen-containing species and residual water, the observed increase nevertheless indicates a significant decrease in oxygen content following irradiation.^35^

Figure 4b and 4c present high-resolution C 1s spectra of pristine and bombarded GO foils, respectively. The spectra were deconvoluted into five components assigned to C=C, C–C, C–O, C=O, and O—C=O states located at 284.4 ± 0.2 eV, 285.0 ± 0.1 eV, 286.3 ± 0.2 eV, 287.8 ± 0.2 eV, and 288.9 ± 0.2 eV, respectively.^36^ The most significant difference between the two samples (see Table 1) is the decrease in the concentration of oxygen-containing functional groups, particularly C–O species, accompanied by a substantial increase in the graphitic C=C contribution, indicating partial restoration of the graphitic sp^2^ carbon network following Cu ion irradiation. Similarly, the O 1s spectra shown in Figure 4d and 4e were deconvoluted into three components associated with C–O, C=O, and O—C=O states located at 533.0 ± 0.2 eV, 532.3 ± 0.2 eV, and 531.5 ± 0.2 eV, respectively. These observations are in good agreement with the Raman spectroscopy and EDS results, which also indicate an increased C/O ratio and partial restoration of graphitic ordering in the bombarded regions.

**Table 1:** Concentration (in At. %) of Individual States Deconvoluted from C 1s and O 1s High-Resolution X-ray Photoelectron Spectra

C 1s					
Sample	C=C	C–C	C–O	C=O	O—C=O
GO Pristine	12.2 ± 0.1	57.2 ± 0.1	24.1 ± 0.1	4.8 ± 0.1	1.7 ± 0.1
GO Bombarded	25.2 ± 0.1	49.9 ± 0.1	18.6 ± 0.1	3.2 ± 0.1	3.1 ± 0.1

O 1s			
Sample	C–O	C=O	O—C=O
GO Pristine	76.9 ± 0.1	15.4 ± 0.1	7.7 ± 0.1
GO Bombarded	75.2 ± 0.1	12.8 ± 0.1	12.0 ± 0.1

To better understand the interaction of energetic Cu ions with GO and the origin of the observed structural modifications, stopping-power simulations were performed, as shown in Figure S1. The simulations indicate that electronic stopping dominates over a substantial portion of the ion trajectory, suggesting that electronic excitation contributes significantly to irradiation-induced deoxygenation and partial restoration of graphitic sp^2^ domains. At larger penetration depths, nuclear stopping becomes increasingly significant, which may promote defect formation, atomic displacement, local sputtering, and thinning of the GO foil.

These observations are consistent with the experimentally observed decrease in oxygen-containing functional groups revealed by XPS and EDS, changes in graphitic ordering observed by Raman spectroscopy, and local height reduction detected by AFM. Therefore, the structural and electrochemical modifications of GO induced by Cu ion bombardment likely result from the combined contributions of electronic excitation and nuclear collision processes.

Although partial implantation of Cu ions cannot be fully excluded, implantation effects are expected to be limited under the present irradiation conditions due to the relatively moderate ion fluence and the intermediate mass of Cu ions. Consequently, the observed structural and electrochemical modifications are more likely dominated by irradiation-induced deoxygenation, defect formation, local thinning, and partial restoration of graphitic domains rather than by retained Cu species.

Following characterization, the bombarded GO foils were examined by using scanning electrochemical microscopy (SECM). In this technique, a microelectrode with a platinum ultramicroelectrode (UME) tip is brought into close proximity to the sample surface, and the faradaic current resulting from a redox mediator in solution is measured.^37^ When the UME is positioned in the bulk electrolyte, a steady-state current governed by diffusion-limited transport of the redox mediator is established.^37^

In this study, SECM was operated in feedback mode, wherein the current response is modulated by the electrochemical properties of the substrate. Two primary feedback regimes are observed. In negative feedback, the approach of the UME to an electrochemically inert or insulating surface leads to suppression of the tip current. This occurs due to hindered diffusion of the redox mediator to the electrode surface as the substrate acts as a physical barrier without facilitating regeneration of the redox species.^37^

In contrast, positive feedback is observed when the substrate is electrochemically active or conductive. As the UME approaches such a surface, the oxidized or reduced form of the redox mediator is regenerated on the substrate and diffuses back to the UME tip. This dynamic redox cycling enhances the local concentration of electroactive species at the electrode interface, thereby increasing the tip current. Such behavior is characteristic of metallic or otherwise conductive materials that support facile electron transfer.^37^

To investigate the electrochemical activity of bombarded GO foils, we selected 5 mM [Ru­(NH_3_)_6_]­Cl_3_ in a 1 M KCl supporting electrolyte. The [Ru­(NH_3_)_6_]­Cl_3_ probe undergoes reduction from Ru^3+^ to Ru^2+^. The [Ru­(NH_3_)_6_]^3+^ /^2+^ couple was selected as a well-established outer-sphere redox mediator whose response is generally considered relatively insensitive to specific surface functional groups or adsorption effects on carbon-based electrodes and instead primarily reflects local electron transfer kinetics and electronic properties of the substrate.^38^ Therefore, the observed SECM contrast is interpreted mainly in terms of irradiation-induced structural and electronic modifications of GO. Ferro/ferricyanide was also evaluated as an alternative redox mediator. However, only weak and spatially diffuse SECM contrast was observed under the present experimental conditions (see Figure S2). In contrast to the [Ru­(NH_3_)_6_]^3+^ /^2+^ mediator, the [Fe­(CN)_6_]^3 –^ /^4 –^ system did not exhibit pronounced edge-localized enhancement and showed substantially weaker electrochemical contrast overall, with no positive current feedback loop. This behavior is consistent with the known sensitivity of the [Fe­(CN)_6_]^3 –^ /^4 –^ redox couple toward surface interactions, adsorption phenomena, and defect structure on carbon materials. Consequently, the present work focuses primarily on the [Ru­(NH_3_)_6_]^3+^ /^2+^ system, whose outer-sphere character enables more direct probing of irradiation-induced changes in local electron transfer behavior and electronic properties of GO.

Figure 5a displays a cyclic voltammogram of [Ru­(NH_3_)_6_]­Cl_3_ obtained prior to the SECM measurements, exhibiting typical diffusion-limited behavior at microelectrodes. Figure 5b shows an SECM scan with the pristine GO region located in the lower part and the bombarded GO stripes in the upper region. To account for potential current baseline shifts caused by electrolyte evaporation (see Experimental and Figure S3a), each approach curve was normalized by dividing the final current value by the initial current value.

**Figure 5:**
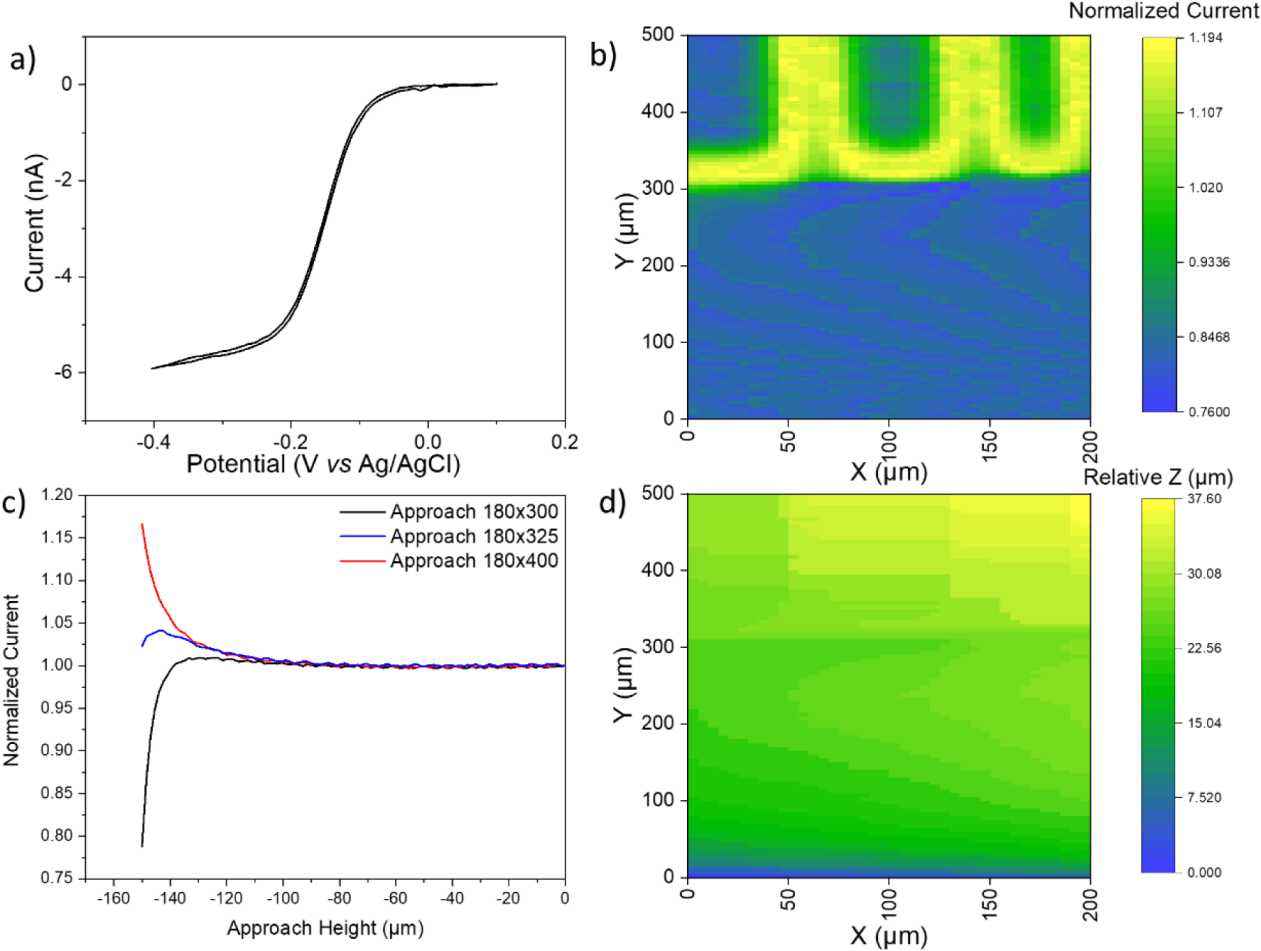
a) Cyclic voltammogram of 5 mM [Ru­(NH_3_)_6_]­Cl_3_ in 1 M KCl recorded at a microelectrode prior to SECM measurements, showing typical diffusion-limited behavior. b) Normalized SECM current map over a GO foil. c) Normalized approach curves at three representative locations: 180 × 300 (pristine GO, blue), 180 × 325 (edge of bombarded stripe, black), and 180 × 400 (bombarded stripe, red). d) Relative Z-height map acquired during the SECM scan.

Figure 5c presents normalized approach curves recorded at three locations: 180 × 300 (pristine GO), 180 × 325 (adjacent to a bombarded stripe), and 180 × 400 (within a bombarded stripe). The curve at 180 × 300 exhibits a clear negative feedback characteristic, consistent with the insulating nature of GO. At 180 × 400, the curve reveals a mixed response—an initial current increase followed by a slight decrease, suggesting partial enhancement. The curve at 180 × 325 shows pronounced positive feedback with significantly elevated current, indicative of increased electrochemical activity near the bombarded region.

Figure 5d shows the relative Z-height map of the GO foil obtained during SECM scanning. A gradual apparent increase in Z-height is observed across the scan, which is attributed to mechanical creep or drift of the tip rather than actual surface topography (see Figure S3b). This interpretation is supported by AFM measurements, which revealed only minor height variations (on the order of several microns over 50 μm).

To distinguish real surface features from drift artifacts, a drift-corrected Z-height map is presented in Figure S3c. This correction highlights that, especially in the central region of the scan (Y = 100–300 μm), surface topography correlates with the electrochemical response. Enhanced current is consistently observed at the regions exhibiting local height discontinuities, supporting the hypothesis, well-documented in the literature, that GO edges are key sites of electrochemical activity.^39,40^

Additionally, the current response appears to correlate with the width of the bombarded stripes, with the narrowest stripe exhibiting the highest activity. We propose that this enhancement is not primarily due to the chemical reduction of GO by Cu ions, but rather due to the increased exposure of GO edges within the bombarded regions. This interpretation is supported by AFM measurements, which show that the bombarded stripes are approximately 1 μm lower in height than the surrounding GO, indicating partial removal or thinning of the GO layer. The resulting edge-rich regions then exhibit enhanced electrochemical activity. Notably, the strongest SECM enhancement was observed near the boundaries of irradiated stripes, whereas the interiors of the bombarded regions exhibited substantially weaker contrast. If the electrochemical response originated solely from uniform conductivity recovery within the irradiated regions, a more homogeneous enhancement across the stripe interiors would be expected. Instead, the localized enhancement near stripe boundaries is consistent with increased contributions from edge-rich and structurally disrupted regions generated during ion bombardment. Nevertheless, contributions from conductivity recovery, defect-mediated charge transport, and local topographical effects cannot be fully excluded.

Elemental mapping by EDS was performed after SECM measurements (Figure S4) to investigate the spatial distribution of key elements across the GO foil. Both carbon and oxygen exhibited similar elemental distributions prior to SECM measurements. Notably, the ruthenium map showed a pronounced depletion of the Ru signal within the bombarded regions while remaining detectable in surrounding areas. This observation implies that Ru species are less likely to accumulate or adsorb onto the bombarded stripes despite those areas exhibiting enhanced electrochemical activity in SECM. The preferential localization of Ru in the unbombarded regions suggests that Ru^3+^ ions likely interact with oxygen-containing functional groups such as carboxyl, hydroxyl, or epoxy moieties on the GO surface. Such behavior is consistent with adsorption not being the dominant origin of the enhanced electrochemical response and instead suggests an important contribution from irradiation-induced structural and electronic modifications of GO.

Finally, the extent of GO modification is expected to depend strongly on ion species, ion energy, and ion fluence, since these parameters determine the balance between electronic stopping, nuclear stopping, sputtering, and defect formation. Lighter ions are expected to favor electronic excitation and more uniform reduction effects, whereas heavier transition-metal ions may induce stronger local damage, thinning, and edge formation due to enhanced nuclear stopping and sputtering.^41^ Similarly, lower ion fluences would likely produce weaker structural and electrochemical modifications, while higher fluences may enhance defect generation and local thinning, potentially at the expense of structural integrity. The stopping simulations presented here further suggest that variations in ion species or irradiation energy could substantially alter the relative contributions of electronic and nuclear energy-loss processes. These observations highlight the potential of ion-beam irradiation as a versatile approach for localized tuning of GO electrochemical and structural properties.

## Conclusions

In this study, we demonstrated that focused Cu ion beam irradiation can be employed to selectively modify the structural and electrochemical properties of graphene oxide (GO) foils. Using a combination of SEM, EDS, Raman spectroscopy, AFM, XPS, and SECM with the [Ru­(NH_3_)_6_]^3+^ redox probe, we observed that ion bombardment leads to partial reduction of GO, as evidenced by the removal of oxygen functionalities, increased C/O ratio, and enhanced graphitic character.

SECM measurements revealed a substantial enhancement of electrochemical activity in the bombarded regions, especially at and near the GO edges. The enhanced electrochemical response is consistent with combined contributions from local thinning, defect formation, partial restoration of graphitic conductivity, and increased exposure of edge-rich regions generated during ion bombardment. Importantly, EDS elemental mapping revealed lower Ru accumulation in the irradiated regions, which is consistent with the enhanced electrochemical response being dominated by irradiation-induced structural and electronic modifications of GO rather than adsorption effects alone.

Moreover, the long-range nature of the edge influence, extending into nearby unbombarded areas, suggests that GO edge sites play a pivotal role in modulating the local electron transfer dynamics. These findings underline the effectiveness of Cu ion beams for site-specific tuning of GO electrochemical properties, offering a valuable platform for nanoscale patterning and functionalization in sensing or energy-related applications.

## Experimental Section

### GO Synthesis

A solution of concentrated sulfuric acid (360 mL) and phosphoric acid (40 mL) in a 9:1 volume ratio was first cooled to below 0 °C. Finely ground graphite (3.0 g) was added, followed by the slow addition of potassium permanganate (18.0 g). The highly exothermic oxidation raised the temperature to 20–25 °C. The suspension was stirred and subsequently heated to 50 °C for 12 h to complete the reaction.

After cooling to ∼20 °C, the mixture was poured onto crushed ice containing 3 mL of 30% H_2_O_2_ to quench residual permanganate and MnO_2_. When the ice had melted, additional 30% H_2_O_2_ was added dropwise until the solution tested negative for permanganate and manganese dioxide.

The resulting graphite oxide was purified by repeated centrifugation and redispersion in deionized water until sulfate ions were no longer detectable. Finally, the graphite oxide slurry was dried under vacuum.

### GO Foil Preparation

Graphene oxide foils were prepared by the vacuum filtration of a well-dispersed aqueous graphene oxide suspension through a membrane filter. After complete filtration, the resulting thin, uniform films were carefully peeled off from the membrane once they were dry. The freestanding graphene oxide foils were flexible, self-supporting, and ready for further characterization and processing.

### Ion Beam Modification

Ion-beam lithography was carried out in an “Oxford Microbeams” microbeam chamber using 2.5 MeV Cu^4+^ ions with a beam current of 3–5 pA. The beam was focused to a spot size of 20 × 60 μm^2^ on the surface of the graphene oxide foil. Irradiation was performed at a fluence of 1 × 10^1 4^ cm^–2^ (640 nC mm^–2^). A microstructured pattern was created as a 30 × 30 pixel image (600 × 600 μm^2^) with a pixel size of 20 μm. Line widths ranged from 20 to 60 μm, and the interline spacing was fixed at 60 μm. Identical foils were also uniformly irradiated across the entire surface under the same conditions (2.5 MeV Cu^4+^ ions, 1 × 10^1 4^ cm^–2^ fluence) to serve as reference samples for XPS analysis, where a broader modified area was required. During irradiation, the ion current density was maintained at 5 nA·cm^–2^.

### Raman Spectroscopy

Raman spectra were acquired using a Renishaw InVia Raman microscope equipped with a 532 nm excitation laser. Measurements were performed under ambient conditions using a laser power below 1 mW to avoid thermal damage of the GO foil. A 50× objective was used for focusing, and the spectral resolution was better than 1 cm^–1^.

Raman measurements were collected from multiple locations within pristine GO regions, Cu-ion bombarded stripes, and crack regions adjacent to the irradiated areas in order to evaluate spatial homogeneity and reproducibility of the irradiation-induced structural modifications. The reported ID/IG ratios represent the average values obtained from these measurements.

### Electron Microscopy and EDS

Scanning electron microscopy (SEM) and energy-dispersive X-ray spectroscopy (EDS) were carried out by using a Tescan MAIA3 microscope operated at an acceleration voltage of 10 kV. Samples were fixed onto SEM stubs by using carbon tape and analyzed under high-vacuum conditions. EDS elemental mapping was performed to assess the distribution of carbon, oxygen, and ruthenium across the GO foil, following SECM experiments. These measurements were conducted on samples that had previously undergone electrochemical analysis in order to correlate the morphology and elemental composition with electrochemical activity.

### X-ray Photoelectron Spectroscopy (XPS)

X-ray photoelectron spectroscopy (XPS) measurements were conducted using a Phoibos 100 spectrometer (Specs, Germany) equipped with a monochromatic Al Kα X-ray source (1486.7 eV). Survey spectra were acquired with a pass energy of 50 eV and a step size of 1 eV, while high-resolution spectra were recorded with a pass energy of 20 eV and a step size of 0.1 eV.

To evaluate the chemical composition changes induced by Cu ion irradiation, both pristine GO foils and GO foils irradiated uniformly over a larger area (∼1 cm^2^) under identical irradiation conditions were analyzed. The larger-area irradiated samples were prepared to enable reliable XPS characterization, as the microstructured irradiated stripes used for SECM measurements were below the spatial resolution limit of the XPS instrument.

Charge compensation was performed using a flood gun, and all spectra were calibrated using the graphitic C 1s component at 284.8 eV. High-resolution C 1s and O 1s spectra were analyzed by peak deconvolution using mixed Gaussian–Lorentzian functions after Shirley background subtraction.

### Atomic Force Microscopy (AFM)

Atomic force microscopy (AFM) characterization was carried out using an NT-MDT Ntegra Spectra instrument in tapping mode. The bombarded GO foil was mounted onto a metallic stub before measurement.

### Scanning Electrochemical Microscopy (SECM)

Scanning electrochemical microscopy (SECM) measurements were performed on a GO foil irradiated with Cu ions and subsequently mounted on an Au/Si/SiO_2_ substrate using conductive carbon tape. Initial characterization involved cyclic voltammetry (CV) in an electrolyte solution containing 5 mM [Ru­(NH_3_)_6_]­Cl_3_ in 1 M KCl. A 10 μm Pt microelectrode served as the working electrode, with Ag/AgCl and Pt wire used as the reference and counter electrodes, respectively.

From the CV response, a bias of −0.4 V vs Ag/AgCl was selected for approach curve measurements. Each approach was terminated when the current deviated by more than 15% from its initial value, indicating proximity to the surface. Following the first approach curve, the probe was retracted vertically by 150 μm. The array scan was then performed by sequentially repeating approach curves across the sample surface, with a lateral step size of 5 μm in both the X and Y directions, while keeping all other parameters constant.

SECM approach curve data from array scans were processed to extract spatially resolved electrochemical and topographical information. For current heatmaps, the final current value of each individual approach curve was normalized to its initial current to account for baseline drift caused by electrolyte evaporation or tip fouling. This normalization allowed for the comparison of electrochemical activity across the sample, independent of absolute current magnitude. The resulting normalized current values were then mapped as a function of their respective X and Y coordinates, with a 5 μm step size, to generate two-dimensional activity maps.

For height heatmaps, the Z position corresponding to the end point of each approach curve (typically where a 15% change in current occurred) was used to estimate surface topography. The relative Z value was plotted against the X and Y coordinates to produce a spatial height profile. To account for the instrumental drift during scanning, a detrending correction was applied. This was done by fitting and subtracting the global drift trend (based on the measurement index vs average Z) from the raw height data. The corrected height map revealed local morphological features that were otherwise obscured by systematic drift.

All data processing was carried out using custom Python scripts employing NumPy, pandas, and Matplotlib libraries. Where relevant, approach curves were selected and plotted individually to illustrate distinct electrochemical behaviors, such as negative or positive feedback, observed across different regions of the GO foil.

### Stopping and Range of Ions in Matter

The projected range and energy losses of the 2.5 MeV Cu^4+^ ions in the GO were estimated by the SRIM full-cascade Monte Carlo simulation.^42^ The composition of the as-prepared GO sample used for the SRIM simulation was determined by EDS and XPS. The density of the substance was determined to be 1.36 g·cm^–3^ by microbalance weighing.

## Supplementary Material



## Data Availability

The data generated during and/or analyzed during the study are accessible via the Zenodo repository: https://zenodo.org/records/16268856.

## Notes

Stopping and range of ions in matter simulations, scanning electrochemical microscopy measurements of [Fe­(CN)_6_]^3 –^ /^4 –^ redox couple, baseline current vs measurement index, height evolution vs measurement index, and corrected height profile after drift correction for [Ru­(NH_3_)_6_]^3+^ /^2+^ redox couple, SEM image after SECM measurements, and corresponding elemental maps. The following files are available free of charge (PDF)

## References

[1] Novoselov K. S., Geim A. K., Morozov S. V., Jiang D.-E., Zhang Y., Dubonos S. V., Grigorieva I. V., Firsov A. A. (2004). Electric field effect
in atomically thin carbon films. Science.

[2] Chung C., Kim Y.-K., Shin D., Ryoo S.-R., Hong B. H., Min D.-H. (2013). Biomedical applications of graphene and graphene oxide. Acc. Chem. Res..

[3] Smith A. T., LaChance A. M., Zeng S., Liu B., Sun L. (2019). Synthesis,
properties, and applications of graphene oxide/reduced graphene oxide
and their nanocomposites. Nano Mater. Sci..

[4] Huang X.-M., Liu L.-Z., Zhou S., Zhao J.-J. (2020). Physical properties
and device applications of graphene oxide. Front.
Phys..

[5] Chen D., Feng H., Li J. (2012). Graphene oxide:
preparation, functionalization,
and electrochemical applications. Chem. Rev..

[6] Roy S., Soin N., Bajpai R., Misra D., McLaughlin J. A., Roy S. S. (2011). Graphene oxide for
electrochemical sensing applications. J. Mater.
Chem..

[7] Malinský P., Macková A., Mikšová R., Kováčiková H., Cutroneo M., Luxa J., Bouša D., Štrochová B., Sofer Z. (2017). Graphene oxide layers
modified by light energetic ions. Phys. Chem.
Chem. Phys..

[8] Yu W., Sisi L., Haiyan Y., Jie L. (2020). Progress in the functional
modification of graphene/graphene oxide: A review. RSC Adv..

[9] Agarwal V., Zetterlund P. B. (2021). Strategies
for reduction of graphene oxide –
A comprehensive review. Chem. Eng. J..

[10] Liu Z., Navik R., Tan H., Xiang Q., Wahyudiono, Goto M., Ibarra R. M., Zhao Y. (2022). Graphene-based
materials prepared by supercritical fluid technology
and its application in energy storage. J. Supercrit.
Fluids.

[11] Pei S., Cheng H.-M. (2012). The reduction of
graphene oxide. Carbon.

[12] Cutroneo M., Havranek V., Mackova A., Malinsky P., Torrisi L., Lorincik J., Luxa J., Szokolova K., Sofer Z., Stammers J. (2019). Localized deoxygenation
of graphene
oxide foil by ion microbeam writing. Vacuum.

[13] Lobo D. E., Fu J., Gengenbach T., Majumder M. (2012). Localized deoxygenation and direct
patterning of graphene oxide films by focused ion beams. Langmuir.

[14] Lehtinen O., Kotakoski J., Krasheninnikov A. V., Tolvanen A., Nordlund K., Keinonen J. (2010). Effects of
ion bombardment on a two-dimensional target:
Atomistic simulations of graphene irradiation. Phys. Rev. B.

[15] Olejniczak A., Nebogatikova N. A., Frolov A. V., Kulik M., Antonova I. V., Skuratov V. A. (2019). Swift heavy-ion
irradiation of graphene oxide: Localized
reduction and formation of sp-hybridized carbon chains. Carbon.

[16] Bai Z., Zhang L., Liu L. (2015). Bombarding graphene with oxygen ions:
combining effects of incident angle and ion energy to control defect
generation. J. Phys. Chem. C.

[17] Vázquez H., Åhlgren E. H., Ochedowski O., Leino A. A., Mirzayev R., Kozubek R., Lebius H., Karlušic M., Jakšic M., Krasheninnikov A. V. (2017). Creating nanoporous
graphene with swift heavy ions. Carbon.

[18] Rosy, Singh F., Goyal R. N. (2015). Structural
and electrochemical characterization of carbon ion beam irradiated
reduced graphene oxide and its application in voltammetric determination
of norepinephrine. RSC Adv..

[19] Kumar S., Tripathi A., Singh F., Khan S. A., Baranwal V., Avasthi D. K. (2014). Purification/annealing of graphene with 100-MeV Ag
ion irradiation. Nanoscale Res. Lett..

[20] Hareesh K., Joshi R. P., Shateesh B., Asokan K., Kanjilal D., Late D., Dahiwale S., Bhoraskar V., Haram S., Dhole S. (2015). Reduction of graphene oxide by 100
MeV Au ion irradiation and its application as H2O2 sensor. J. Phys. D: Appl. Phys..

[21] Saravanan K., Jayalakshmi G., Suresh K., Sundaravel B., Panigrahi B. K., Phase D. M. (2018). Structural evaluation of reduced
graphene oxide in graphene oxide during ion irradiation: X-ray absorption
spectroscopy and in-situ sheet resistance studies. Appl. Phys. Lett..

[22] Jayalakshmi G., Saravanan K., Panigrahi B., Sundaravel B., Gupta M. (2018). Tunable electronic,
electrical and optical properties of graphene
oxide sheets by ion irradiation. Nanotechnology.

[23] Saravanan K., Sundaravel B., Panigrahi B. K. (2018). Resonant Rutherford backscattering
spectrometric analysis on ion beam reduced graphene oxide. AIP Conf. Proc..

[24] Jayalakshmi G., Saravanan K., Arun T., Suresh K., Sundaravel B., Panigrahi B., Kanjilal D. (2017). Structure and electron field emission
properties of ion beam reduced graphene oxide sheets. Carbon.

[25] Saravanan K., Panigrahi B., Suresh K., Sundaravel B., Magudapathy P., Gupta M. (2018). A novel green approach for reduction
of free standing graphene oxide: electrical and electronic structural
investigations. Nanotechnology.

[26] Guex L. G., Sacchi B., Peuvot K. F., Andersson R. L., Pourrahimi A. M., Ström V., Farris S., Olsson R. T. (2017). Experimental
review: chemical reduction of graphene oxide (GO) to reduced graphene
oxide (rGO) by aqueous chemistry. Nanoscale.

[27] Botas C., Álvarez P., Blanco C., Santamaría R., Granda M., Gutiérrez M. D., Rodríguez-Reinoso F., Menéndez R. (2013). Critical temperatures
in the synthesis of graphene-like
materials by thermal exfoliation–reduction of graphite oxide. Carbon.

[28] Tyagi C., Khan S. A., Sulania I., Meena R., Avasthi D. K., Tripathi A. (2018). Evidence of ion-beam-induced annealing
in graphene
oxide films using in situ X-ray diffraction and spectroscopy techniques. J. Phys. Chem. C.

[29] Gawlik G., Ciepielewski P., Jagielski J., Baranowski J. (2017). Modification
of graphene by ion beam. Nucl. Instrum. Methods
Phys. Res., Sect. B.

[30] Cutroneo M., Havranek V., Mackova A., Malinsky P., Torrisi L., Silipigni L., Fazio B., Torrisi A., Szokolova K., Sofer Z. (2019). Effects of the ion bombardment
on the structure and
composition of GO and rGO foils. Mater. Chem.
Phys..

[31] López-Díaz D., Lopez Holgado M., García-Fierro J. L., Velázquez M. M. (2017). Evolution
of the Raman spectrum with the chemical composition of graphene oxide. J. Phys. Chem. C.

[32] Lopez-Diaz D., Delgado-Notario J. A., Clericò V., Diez E., Merchan M. D., Velázquez M. M. (2020). Towards
understanding the Raman spectrum of graphene
oxide: the effect of the chemical composition. Coatings.

[33] Dresselhaus M. S., Jorio A., Hofmann M., Dresselhaus G., Saito R. (2010). Perspectives on Carbon Nanotubes and Graphene Raman Spectroscopy. Nano Lett..

[34] Ferrari A. C. (2007). Raman spectroscopy
of graphene and graphite: Disorder, electron–phonon coupling,
doping and nonadiabatic effects. Solid State
Commun..

[35] Liu R., Gong T., Zhang K., Lee C. (2017). Graphene oxide papers
with high water adsorption capacity for air dehumidification. Sci. Rep..

[36] Jankovský O., Nováček M., Luxa J., Sedmidubský D., Fila V., Pumera M., Sofer Z. (2016). A New Member
of the
Graphene Family: Graphene Acid. Chem. –
Eur. J..

[37] Kwak J., Bard A. J. (1989). Scanning electrochemical microscopy. Theory of the
feedback mode. Anal. Chem..

[38] Chen P., McCreery R. L. (1996). Control of Electron
Transfer Kinetics at Glassy Carbon
Electrodes by Specific Surface Modification. Anal. Chem..

[39] Yuan W., Zhou Y., Li Y., Li C., Peng H., Zhang J., Liu Z., Dai L., Shi G. (2013). The edge-
and basal-plane-specific electrochemistry of a single-layer graphene
sheet. Sci. Rep..

[40] Song Y., Feng M., Zhan H. (2015). Geometry-dependent electrochemistry
of graphene oxide family. Electrochem. Commun..

[41] Malinský P., Macková A., Florianová M., Cutroneo M., Hnatowicz V., Boháčová M., Szőkölová K., Böttger R., Sofer Z. (2019). The Structural and Compositional
Changes of Graphene Oxide Induced by Irradiation With 500 keV Helium
and Gallium Ions. Phys. Status Solidi B.

[42] Ziegler, J. F. ; Biersack, J. P. The stopping and range of ions in matter. In Treatise on heavy-ion science: volume 6: Astrophysics, chemistry, and condensed matter; Springer, 1985; pp. 93–129.

