# Supplementary material for: Edge Exposure and Electrochemical Modulation of Graphene Oxide by Focused Cu Ion Bombardment
Source: Langmuir. 2026 Jun 23;42(26):18801–9. doi: 10.1021/acs.langmuir.6c01117 (PMC13352623; doi:10.1021/acs.langmuir.6c01117)
Supplement: Supplementary file 1 [file la6c01117_si_001.pdf]

Edge Exposure and Electrochemical Modulation of Graphene Oxide by Focused Cu Ion Bombardment

Jan Luxa,[a] Petr Malinský,[b,c] Josef Novák,[b,c] Vladimír Havránek,[b] Vlastimil Mazánek,[a] Jakub Regner[a] and Zdeněk Sofer[a]*

a Department of Inorganic Chemistry, University of Chemistry and Technology Prague, Technická 5, 166 28 Prague 6, Czech Republic

b Nuclear Physics Institute of the Czech Academy of Sciences, Hlavní 130, 250 68 Řež, Czech Republic

c Department of Physics, Faculty of Science, University of J. E.Purkyně, 400 96 Usti nad Labem, Czech Republic

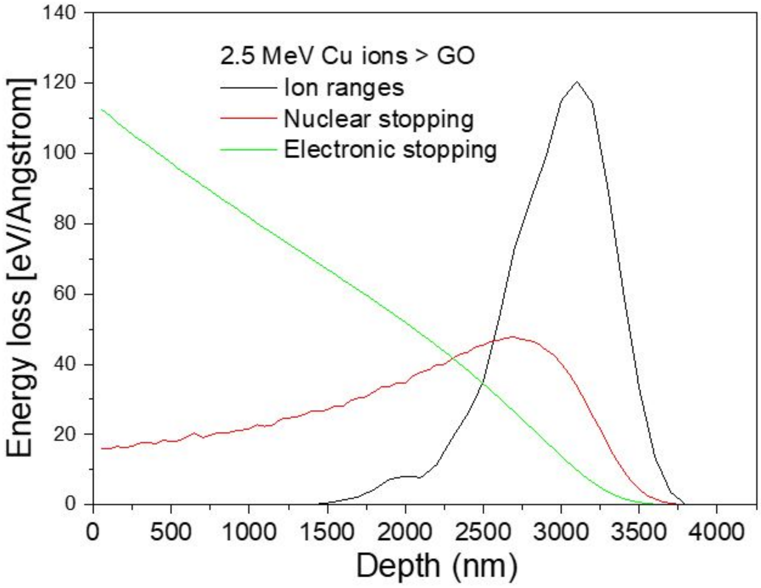

Figure S1. SRIM stopping simulation of 2.5 MeV Cu ions in graphene oxide showing electronic stopping, nuclear stopping, and ion range distribution.

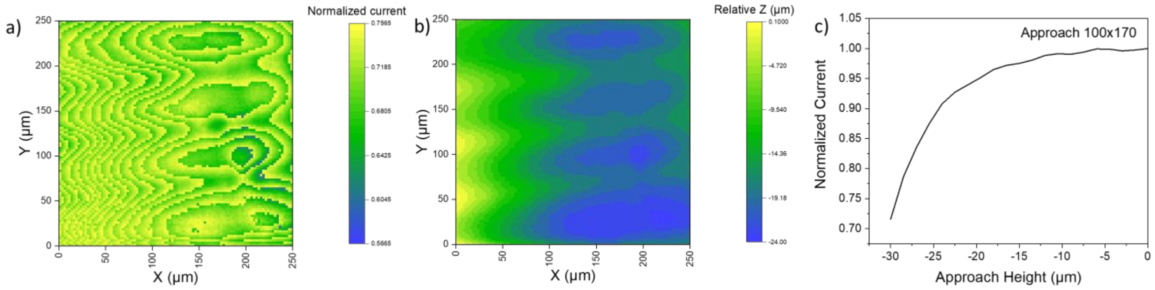

Figure S2. a) Normalized SECM current map acquired using 5 mM $Fe(CN)_6^{3-}/^{4-}$, b) relative Z-height map reconstructed from SECM approach-curve analysis acquired simultaneously with the current map and c) normalized approach curve at 100x170

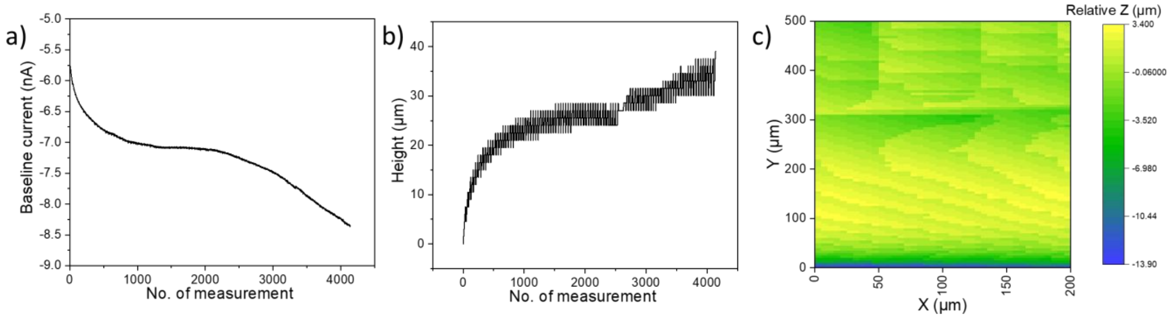

Figure S3. a) Baseline current vs number of measurement, b) height evolution as vs number of measurement, c) corrected height profile after drift correction.

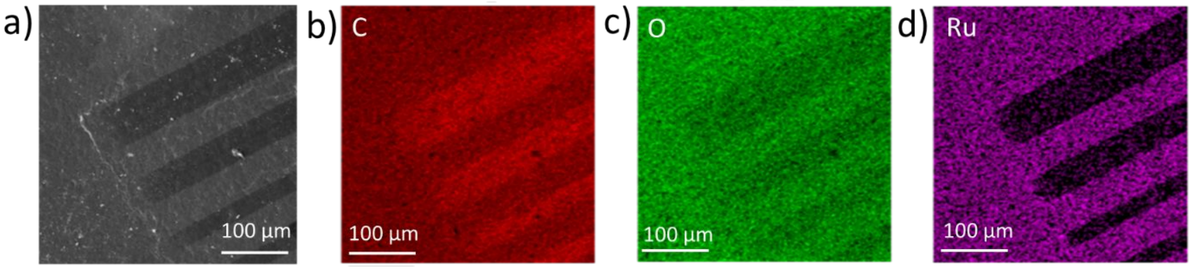

Figure S4. a) SEM image after SECM measurements, b) carbon elemental map, c) oxygen elemental map and d) ruthenium elemental map.
